# Association between quarter-level milk somatic cell count and intramammary bacterial infection in late-lactation Irish grazing dairy cows

**DOI:** 10.3168/jdsc.2022-0317

**Published:** 2023-03-16

**Authors:** Ainhoa Valldecabres, Clare Clabby, Pat Dillon, Pablo Silva Boloña

**Affiliations:** Teagasc, Animal and Grassland Research and Innovation Centre, Moorepark, Fermoy, Co. Cork, Ireland P61 C996

## Abstract

The objectives of our study were to describe quarter-level prevalence of intrammamary infection (IMI), to evaluate the performance of commonly used somatic cell count (SCC) thresholds for the diagnosis of quarter-level IMI, and to determine those with maximized sensitivity (Se) and specificity (Sp) for identifying quarter-level IMI as defined by positive aerobic culture in late-lactation grazing dairy cows. In this observational study, quarter milk samples were collected from all cows in 21 commercial spring-calving, pasture-based Irish dairy herds. Total SCC determination and aerobic bacterial culture were performed in 8,177 quarter milk samples obtained between 238 and 268 d in milk from 465 primiparous and 1,609 multiparous cows. The Se and Sp of SCC thresholds used for diagnosis of IMI were evaluated against the gold standard aerobic culture separately for all, primiparous, and multiparous cows. The overall prevalence of bacteriologically infected quarters was 6.3%, and it was higher among primiparous (11.3%) than multiparous cows (5.5%). However, considering all samples, quarter-level SCC was higher for multiparous than for primiparous cows (195,250 ± 21,422 vs. 115,940 ± 26,260 cells/mL). Associated Se and Sp for the 200,000 cells/mL threshold were 59.2% and 88.0% for all, 52.7% and 95.4% for primiparous, and 62.9% and 85.9% for multiparous cows, respectively. Receiver operating characteristic curve analyses determined the thresholds that optimized the Se and Sp of a positive bacterial culture: 101,000 cells/mL for all cows [Se = 80.0%; Sp = 76.4%; area under the curve (AUC) = 0.84], 61,000 cells/mL for primiparous (Se = 87.1%; Sp = 84.0%; AUC = 0.90), and 101,000 cells/mL for multiparous (Se = 80.9%; Sp = 72.6%; AUC = 0.83). The results indicate that the 200,000 cells/mL threshold was inefficient in identifying late-lactation quarter-level IMI (low Se) in the studied herds where the main etiological agent was Staphylococcus aureus. Suggested quarter-level SCC thresholds have the potential of serving as a supporting tool for dry cow therapy decisions and warrant further study in late-lactation cows from spring-calving, pasture-based herds with S. aureus as the main pathogen causing IMI.

## Highlights

• Quarter-level IMI discrimination was not maximized at the 200,000 cells/mL threshold.
• A 61,000 cells/mL threshold had >80% sensitivity and specificity in primiparous cows.
• A 101,000 cells/mL threshold had >80% sensitivity and >70% specificity in multiparous cows.

## Graphical Abstract

**Figure:**
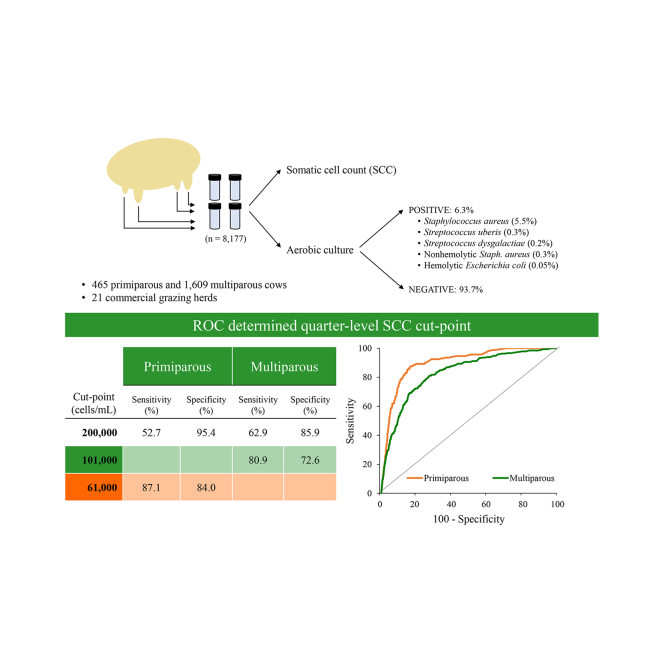
**Summary:** The aims of this study were to describe quarter-level prevalence of intramammary infection (IMI) and somatic cell count (SCC), to evaluate the performance of commonly used SCC thresholds for the diagnosis of quarterlevel IMI and determine alternative thresholds for diagnosis of quarter-level IMI as defined by a positive aerobic culture in late lactation. A total of 8,177 quarter-level milk samples were available for analysis from both primiparous and multiparous cows from 21 Irish herds. The overall prevalence of quarter-level IMI was 6%; higher for primiparous (10.2%) than multiparous (5.2%). The most common etiological IMI agent was *Staphylococcus aureus*. Sensitivity and specificity were maximized at SCC thresholds lower than the traditional 200,000 cells/mL (61,000 cells/mL for primiparous; 101,000 cells/mL for multiparous). The identified quarterlevel SCC thresholds should be evaluated as guidance for selective dry cow therapy in the Irish dairy production scenario.

Cow composite milk samples are often collected for SCC determination, as a proxy for IMI diagnosis and mastitis control throughout lactation, and can be used as a guidance for selective dry cow therapy (**SDCT**). Increased SCC is commonly associated with IMI, defined as a positive bacterial culture (gold standard; Dohoo et al., 1981). However, the magnitude of the increase in SCC varies according to the pathogen causing the infection and other non-cow-level factors such as the dilution of the sample (composite vs. quarter-level sample; Djabri et al., 2002).

*Staphylococcus aureus* causes most of the IMI in Irish dairy herds (Barrett et al., 2005; Keane et al., 2013). This pathogen is a well-known contagious bacteria that is transmitted from infected to uninfected quarters and cows, commonly causing chronic infections and being the most frequent cause of IMI at dry-off (Bradley and Green, 2004). *Staphylococcus aureus* has shown the ability to interfere with the immune response of the udder, which can result in a somewhat less pronounced increase in milk SCC when compared with other pathogens (*Streptococcus uberis* and *Streptococcus agalactiae*; Djabri et al., 2002). The cow udder is composed of 4 independent mammary glands (quarters) so that pathogens have to enter and colonize each of the quarters to cause infection. Consequently, IMI is generally presented in a single quarter. Therefore, it is not surprising that sensitivity (**Se**; ability to correctly identify IMI) and specificity (**Sp**; ability to correctly identify absence of IMI) associated with different SCC thresholds are generally higher for SCC determinations performed at the quarter level, compared with those performed in composite samples (Bach et al., 2019).

In line with the National Mastitis Council (2001) recommendations, a 200,000 cells/mL threshold, which holds practical value due to its reduced diagnostic error (Schukken et al., 2003), is commonly used as an indicator of IMI at the cow or quarter level in primiparous or multiparous cows in Ireland. Other thresholds suggested are 100,000 cells/mL for quarter-level samples (International Dairy Federation, 2013), 120,000 cells/mL for primiparous, and 150,000 cells/mL for multiparous cows (cow-level samples; DairyNZ, 2012).

To the best of the authors' knowledge, no study has evaluated the association between quarter-level SCC and IMI in Ireland or herds where the majority of infections are caused by *S. aureus*. Thus, the objectives of the present observational study were to describe the quarter-level prevalence of IMI and SCC, to determine the effectiveness of the traditional 200,000 cells/mL SCC threshold and others suggested by institutions of reference (100,000 cells/mL for quarter-level samples, 120,000 cells/mL for primiparous, and 150,000 cells/mL for multiparous) to identify quarter-level IMI, and to determine the threshold with maximized Se and Sp for identifying quarter-level IMI in a cohort of late-lactation cows in spring-calving, pasture-based Irish herds.

This study was approved by the Teagasc Animal Ethics Committee (license no. 1542017). All-herd quarter milk samples were collected from a convenient sample of 21 commercial spring-calving grazing herds in Ireland in late lactation (n = 2,074 cows). Irish spring-calving, pasture-based herds are characterized by a compact calving season that coincides with the onset of the grass growing season. In this system, cows are generally out to pasture for the majority of the year and housed during the winter and dry period. The interquartile range (**IQR**) for enrolled herds' size was 66 to 122 cows (national average = 91 cows; Dillon et al., 2021). At the time of sample collection, average bulk tank milk SCC of the herds was 129,890 cells/mL (IQR = 103,400 to 147,500 cells/mL; milk processing company data from the last pick up before study sampling). Whole-lactation cow milk production based on milk supplied for the study herds was on average 6,246 kg (national average = 5,744 kg; Dillon et al., 2021). Parity distribution in the study cohort was 22.4% first, 19.3% second, 19.2% third, 13.1% fourth, and 26.0% ≥fifth. The IQR for cows' DIM at quarter sampling was 238 to 268 d. Quarter sampling was performed on average 35 d before dry-off (IQR = 23 to 44 d). The quarter milk samples were aseptically collected before milking from November to December 2020 by Teagasc research personnel as described by Clabby et al. (2022). The milk was collected into 30-mL sterile milk collection vials without preservative until it reached roughly half of their capacity (15 mL). Samples were transported to the laboratory after collection and frozen at −20°C for analysis upon sampling completion. Within a month of collection, samples were thawed and analyzed for total SCC and presence of bacteria. Somatic cell counts were performed by flow cytometry (Bentley Somacount 300; Bentley Instrument Inc.). Milk samples were cultured using aerobic procedures recommended in the Laboratory Handbook on Bovine Mastitis (National Mastitis Council, 2017). Nonselective blood agar plates were divided into 4 equal quadrants, one for each quarter of the same cow (Oxoid Blood Agar Base No. 2 with 0.1% esculin; Thermo Fisher Scientific). Quarters were considered infected if at least 6 colonies (600 cfu/mL) were identified in the platted quadrant after 24 to 48 h of incubation. If 2 types of colonies were identified on the same plate, the predominant colony was considered the cause of IMI. A sample was considered contaminated if >2 types of colonies were identified on the plate. Only samples with SCC determination and aerobic culture performed were included in the study.

Data summarization and analyses were conducted with SAS (version 9.4; SAS Institute Inc.) unless otherwise stated. Linear and logistic models were used to evaluate the effect of parity (primiparous vs. multiparous) on quarter-level SCC and prevalence of infection (MIXED and GENMOD procedures, respectively). Random effects were added at the herd level to account for clustering of cows within herds and at the cow level to account for clustering of quarters within cows. To comply with assumptions of the analysis and facilitate results interpretation, *P*-values for the SCC comparison were obtained from models using log_10_ SCC, whereas estimates were obtained from analysis using untransformed SCC data. Results are presented as least squares means ± standard error of the mean unless otherwise stated. Using aerobic culture results as the gold standard, Se, Sp, positive (**PPV**), and negative predictive (**NPV**) values were determined for identification of quarter-level IMI based on SCC for a 200,000 cells/mL and a 100,000 cells/mL threshold; for all, primiparous, and multiparous cows; for a 120,000 cells/mL threshold for primiparous cows; and for a 150,000 cells/mL threshold for multiparous cows. The SCC threshold that optimized Se and Sp for identification of quarter-level IMI was determined by receiver operating characteristic curve analyses and maximized area under the curve (**AUC**), weighing false-positive and false-negative results equally, and separately for all, primiparous, and multiparous cows using MedCalc (version 20.110; MedCalc Software Ltd.).

Among the 2,074 cows sampled (465 primiparous and 1,609 multiparous), 92 cows had a missing sample from 1 quarter (either the quarter sample was contaminated, the quarter was not producing milk, or it was being treated) and 1 cow had a missing sample from 2 quarters. Culture was not performed from 1 quarter sample, and insufficient milk volume for SCC determination was collected from 24 quarters. Consequently, the total number of samples available for analyses with quarter SCC and bacterial culture results was 8,177 (1,832 from primiparous and 6,609 from multiparous).

Of all the sampled quarters, 6.3% were infected (n = 515/8,177). Cows had 1 (14.3%; n = 297/2,074), 2 (3.6%; n = 74/2,074), 3 (0.8%; n = 16/2,074), or 4 (0.3%; n = 6/2,074) infected quarters. A higher quarter-level prevalence of IMI was observed among primiparous (11.3%; 95% CI = 9.4 to 13.5%) compared with multiparous cows (5.5%; 95% CI = 4.8 to 6.3%; *P* < 0.001). However, the proportion of multiparous cows' quarter-level samples with SCC ≥200,000 cells/mL (20.1%; 95% CI = 18.3 to 22.0%) was higher than that for primiparous cows (10.5%; 95% CI = 8.6 to 12.8%; *P* < 0.001). The prevalence of cows with at least one quarter infected was 19.0% for all (n = 393/2,074), 29.3% for primiparous (n = 136/465), and 16.0% for multiparous cows (n = 257/1,609). Isolated microorganisms in this study included *Staphylococcus aureus* (5.5%; 450/8,177), *Streptococcus uberis* (0.3%; 23/8,177), *Streptococcus dysgalactiae* (0.2%; 16/8,177), NAS (0.3%; 24/8,177), and non-hemolytic *Escherichia coli* (0.05%; 4/8,177).

Descriptive statistics showed that median quarter-level SCC was 30,000 cells/mL for infected and uninfected quarters from all cows (IQR = 13,000 to 113,000 cells/mL), 18,000 cells/mL for primiparous cows (IQR = 7,000 to 52,000 cells/mL), and 40,000 cells/mL for multiparous cows (IQR = 16,000 to 128,000 cells/mL). Considering all samples, statistical analyses showed that quarter-level SCC was higher for multiparous than for primiparous cows (195,250 ± 21,422 vs. 115,940 ± 26,260 cells/mL; *P* < 0.001), and for quarters with IMI than for uninfected quarters (849,580 ± 34,468 vs. 133,140 ± 20,221 cells/mL; *P* < 0.001). Quarter-level SCC in infected quarters was higher for multiparous than for primiparous cows (*P* < 0.001). Within parity groups, quarter-level SCC was also higher for quarters with IMI compared with the uninfected quarters [primiparous: 580,540 ± 43,059 vs. 58,811 ± 21,036 cells/mL (*P* < 0.001); multiparous: 1,009,900 ± 43,281 vs. 153,790 ± 23,365 cells/mL (*P* < 0.001)].

Values for Se, Sp, PPV, and NPV for the previously mentioned SCC thresholds (100,000, 120,000, 150,000, and 200,000 cells/mL) for all cows (primiparous and multiparous combined) and primiparous and multiparous cows are presented in Table 1. Optimal quarter-level SCC thresholds differed by parity group. The 101,000 cells/mL quarter-level SCC threshold maximized the Se and Sp for all cows [Se = 80.0% (95% CI = 76.3–83.4); Sp = 76.4% (95% CI = 75.4–77.4); PPV = 18.6% (95% CI = 17.7–19.5); NPV = 98.3% (95% CI = 98.0–98.5); AUC = 0.84 (95% CI = 0.83–0.85)] and multiparous cows alone [Se = 80.9% (95% CI = 76.2–85.0); Sp = 72.6% (95% CI = 71.4–73.7); PPV = 14.0% (95% CI = 13.3–14.9); NPV = 98.6% (95% CI = 98.2–98.9); AUC = 0.83 (95% CI = 0.82–0.84)]. The threshold that maximized the Se and Sp for primiparous cows alone was 61,000 cells/mL [Se = 87.1% (95% CI = 81.4–91.6); Sp = 84.0% (95% CI = 82.1–85.7); PPV = 38.0% (95% CI = 35.2–41.0); NPV = 98.3% (95% CI = 97.5–98.8); AUC = 0.90 (95% CI = 0.89–0.92)].

**Table 1:** Sensitivity, specificity, positive predictive (PPV), and negative predictive (NPV) values for previously suggested SCC thresholds for diagnosis of quarter-level IMI defined as culture positive for all (n = 2,074), primiparous (n = 465), and multiparous (n = 1,609) cows sampled at late lactation in 21 spring-calving grazing commercial herds in Ireland

Group	Threshold (cells/mL)	Sensitivity,% (95% CI)	Specificity, % (95% CI)	PPV, % (95% CI)	NPV, % (95% CI)
All cows	200,000	59.2	88	24.8	97
		(54.8–63.5)	(87.2–88.7)	(23.1–26.6)	(96.7–97.3)
	100,000	80.2	76.1	18.4	98.3
		(76.5–83.6)	(75.1–77.0)	(17.5–19.3)	(98.0–98.6)
Primiparous	200,000	52.7	95.4	56.6	94.7
		(45.3–60.0)	(94.3–96.4)	(50.2–62.9)	(93.9–95.4)
	120,000	72.6	90.8	47.2	96.7
		(65.6–78.9)	(89.3–92.2)	(42.9–51.6)	(95.9–97.4)
	100,000	78.5	88.6	43.8	97.3
		(71.9–84.2)	(87.0–90.1)	(40.1–47.7)	(96.5–98.0)
Multiparous	200,000	62.9	85.9	19.6	97.7
		(57.4–68.2)	(85.0–86.8)	(18.0–21.3)	(97.4–98.0)
	150,000	72.0	81.2	17.3	98.2
		(66.9–76.8)	(80.2–82.1)	(16.1–18.6)	(97.8–98.4)
	100,000	81.2	72.6	13.9	98.6
		(76.5–85.2)	(71.4–73.7)	(13.2–14.8)	(98.3–98.9)

Quarter-level IMI was diagnosed in 6.3% of the sampled quarters in our study, with the main etiological agent being *S. aureus* (5.5%) for both, primiparous, and multiparous cows. The definition of infection in our study should be considered when interpreting the etiology of IMI. A standard definition of IMI for all pathogens could result in different Se of the milk culture (ranging from 87% for *S. aureus* to <60% for CNS; Dohoo et al., 2011). Therefore, some bacterial species may have been underdiagnosed herein. The etiologic profile was similar for primiparous and multiparous cows [*S. aureus* was the most commonly isolated pathogen in primiparous (9%) and multiparous cows (4%)]. Different etiological pathogens have been reported as cause for first clinical mastitis case among parities, whereas a similar profile has been described for succeeding cases under confined production systems (Hertl et al., 2014). Late-lactation infections described in this study may represent those succeeding infections, as well as chronic infections.

In agreement with our results, Bach et al. (2019) reported a lower SCC response to quarter-level IMI for primiparous compared with multiparous cows. The higher prevalence of IMI found in primiparous cows in our study could be due to the production system or to husbandry practices that have been associated with mastitis risk (e.g., having primiparous and multiparous cows housed and calving together). To our knowledge there have not been reports of infection levels in late lactation in primiparous cows in this production system. A hypothesis for the high IMI prevalence in late-lactation primiparous cows is that for different reasons they may have acquired a higher number of infections, which then became chronic. However, this hypothesis cannot be tested in the present study.

In the current study and in the study of Bach et al. (2019), the receiver operating characteristic curve calculated thresholds with maximized Se and Sp for identification of quarter-level IMI were lower and resulted in a substantial improvement of test (threshold) diagnostic performance compared with 200,000 cells/mL, long considered a threshold with minimized classification error (Table 1; Schukken et al., 2003). Differences between studies on the IMI prevalence, pathogenic profile, farming system, and lactation stage of enrolled cows could explain the discrepancies on the identified diagnostic thresholds and the 200,000 cells/mL threshold performance (Leeflang et al., 2013). Additionally, overtime genetic selection based on SCC could potentially result in a lower SCC response to infection and, therefore, affect the performance of higher SCC thresholds at predicting IMI. For instance, Wellnitz et al. (2010) reported that very low SCC quarters (potentially quarters from cows genetically selected for low SCC) showed a lower SCC increase when challenged with a *E. coli* LPS, compared with what they termed “normal” SCC quarters. However, the potential link between genetic selection based on SCC and the SCC response to IMI has not been evaluated in the literature. Last, laboratorial techniques used for the determination of SCC may also contribute to the variation between studies and should be considered when interpreting SCC thresholds. The use of frozen milk samples may have resulted on lower SCC in our study. Nevertheless, after freezing samples for up to 28 d, Barkema et al. (1997) concluded that the observed small decrease in SCC may have not been fully attributable to freezing. The thresholds identified in our study had an excellent ability to discriminate among cows with and without IMI as indicated by the high AUC (AUC >0.80; Mandrekar, 2010). The similarity between the identified threshold with maximized Se and Sp for all cows in our study (101,000 cells/mL) and the quarter-level threshold suggested by the International Dairy Federation (100,000 cells/mL; International Dairy Federation, 2013) should be noted. The International Dairy Federation threshold is a result of consulting with experts in the field and appears to be based, among others, on the “Guidelines on normal and abnormal raw milk based on SCC and signs of clinical mastitis” document (National Mastitis Council, 2001).

The SCC overlap between infected and noninfected cows has been commonly shown (McDougall et al., 2021). Thus, using SCC thresholds will result in false positive and negative results regardless of the threshold used. Additionally, by looking at the PPV it is possible to see that any test classifying a quarter as infected will result in a large proportion of falsely categorized quarters. While we present the thresholds with maximized Se and Sp in our study cohort, the best threshold may change based on the circumstances for its use and associated costs of false positive or negative results (e.g., contagious vs. environmental bacterial challenges, dry-off vs. lactation use). What seems clear from this study is that different quarter-level SCC thresholds should be used for primiparous and multiparous cows in Irish dairy herds as they have distinct SCC levels when healthy and SCC response when an infection occurs.

In late lactation, SCC thresholds could be used to guide SDCT. In the literature, assigning dry cow treatment (antibiotic or teat sealant) based on the 200,000 cells/mL cow-level SCC threshold had no effect on udder health (Vasquez et al., 2018; McDougall et al., 2022). However, studies in Ireland have shown that using a threshold of 200,000 cells/mL results in higher SCC in cows treated with teat sealant alone compared with antibiotic plus teat sealant (McParland et al., 2019; Clabby et al., 2022). These findings and those of our study suggest that the SCC threshold for SDCT guidance should be revised, at least for this particular population (Irish spring-calving, pasture-based herds). Higher thresholds result in higher NPV, meaning that a large proportion of cows that need an antibiotic will get it. Given the pathogen profile, production system, and results of previous SDCT studies in Irish spring-calving, pasture-based herds, it could be preferable to aim for a maximized Se (identify as many cows infected as possible). Quarter-level SCC could be a faster solution than quarter-level bacteriological cultures, and potentially more accurate, although more labor intensive, than guiding SDCT based on California Mastitis Test results (McDougall et al., 2022). Furthermore, a quarter-level treatment decision approach could further reduce the use of antimicrobials. Further research should look at the effect on antimicrobial use and IMI cure of considering the proposed quarter-level SCC thresholds in SDCT in the Irish dairy production scenario.
